# Dr. Frank T. Cochran

**Published:** 1912-09

**Authors:** 


Dr. Frank T. Cochran, Jefferson, 1872, died at Hudson, N. Y., July 17, aged 60.

